# Type I and II pulmonary atresia with intact ventricular septum in infants: a 10-year experience in initial surgery at one center

**DOI:** 10.1186/s12872-022-02549-1

**Published:** 2022-03-17

**Authors:** Hailong Song, Ziying Chen

**Affiliations:** Department of Cardiac Surgery, the Second Hospital of Hebei Medical University, No. 215, Heping West Road, Shijiazhuang, Hebei China

**Keywords:** Pulmonary atresia with intact ventricular septum, Balloon dilatation of pulmonary valve, Pulmonary valve incision, Transannular patch, Artificial pulmonary valve implantation

## Abstract

**Background:**

To explore the effect of initial surgery for type I and II pulmonary atresia with intact ventricular septum (PA/IVS).

**Methods:**

50 children with type I PA/IVS and 50 with type II PA/IVS who had undergone initial surgery were enrolled. Children with Type I were divided into groups A (n = 25) and B (n = 25). Group A had received BT shunt combined with PDA ligation and balloon dilatation of pulmonary valve, whereas group B had undergone BT shunt combined with PDA ligation and pulmonary valve incision. Children with type II were divided into groups C (n = 25) and D (n = 25). Group C had received BT shunt combined with PDA ligation, right ventricular outflow tract (RVOT) incision and transannular patch. Group D had undergone BT shunt combined with PDA ligation, RVOT incision, transannular patch and artificial pulmonary valve implantation. The differences in mechanical ventilation time, length of ICU stay, mortality rate, tricuspid Z value, tricuspid regurgitation, oxygen saturation, pulmonary regurgitation, McGoon ratio, pulmonary artery transvalvular pressure, survival rate were compared between groups A and B, between groups C and D respectively.

**Results:**

The ventilator assistance time and length of ICU stay were greater in group C than in group D (80.96 ± 8.42 h vs. 65.16 ± 4.85 h, *P* = 0.045; 222.00 ± 11.72 h vs. 162.48 ± 7.91 h, *P* = 0.048). The pulmonary artery transvalvular pressure was significantly higher in group A than in group B at 3, 6, 12, 24 and 36 months after surgery (64.86 ± 4.13 mmHg vs. 53.04 ± 5.64 mmHg, *P* = 0.045; 69.47 ± 1.93 mmHg vs. 55.95 ± 4.04 mmHg, *P* = 0.005; 80.16 ± 3.76 mmHg vs. 73.24 ± 2.34 mmHg, *P* = 0.035; 62.95 ± 5.64 mmHg vs. 48.47 ± 7.44 mmHg, *P* = 0.04; 53.69 ± 4.89 vs. 45.77 ± 3.26, *P* = 0.02). Furthermore, the tricuspid Z value was significantly greater in group B than in group A at 3 and 24 months after surgery (− (1.37 ± 0.04) vs. − (1.43 ± 0.06), *P* = 0.03; − (0.41 ± 0.06) vs. − (0.51 ± 0.11), *P* = 0.02).

**Conclusions:**

The effect of BT shunt combined with PDA ligation and pulmonary valve incision is superior to BT shunt combined with PDA ligation and balloon dilatation of pulmonary valve, and the effect of BT shunt combined with PDA ligation, RVOT incision, transannular patch and artificial pulmonary valve implantation is superior to BT shunt combined with PDA ligation, RVOT incision and transannular patch.

## Background

Pulmonary atresia with intact ventricular septum (PA/IVS) is a type of complex cyanotic congenital heart disease with a low incidence, accounting for only 1% to 3% of congenital heart disease (CHD) [1]; however, it is a critically ill condition with high mortality.

Given a large anatomical variation of PA/IVS, and diverse surgical treatments in each center, there is still no unified standard [2, 3]. Nevertheless, the reasonable choice of the initial surgery method is the key to improving the survival rate, which is also an important factor affecting the prognosis. Most heart centers choose different initial surgery schemes according to the degree of right ventricular development and presence or absence of right-ventricle-dependent coronary circulation (RVDCC). However, due to the complexity and diversity of PA/IVS, the specific initial surgery methods are different.

This retrospective study aimed to investigate the effect of different initial surgical procedures on the prognosis of type I and type II PA/IVS, and to evaluate the therapeutic effect, thereby providing reliable clinical data for optimizing the selection of initial procedures for PA/IVS.

## Methods

### Clinical data and grouping

Using random number table method, we selected 25 cases of children with type I PA/IVS who had been treated by BT shunt combined with PDA ligation and balloon dilatation of pulmonary valve in our hospital from January 2010 to December 2019, and included them in group A; another 25 cases, who had been initially performed with BT shunt combined with PDA ligation and pulmonary valve incision, were enrolled in group B. Meanwhile, 25 cases of children with type II PA/IVS in group C had initially undergone BT shunt combined with PDA ligation, right ventricular outflow tract (RVOT) incision and transannular patch, and another 25 cases were enrolled in group D, in which BT shunt combined with PDA ligation, RVOT incision, transannular patch and artificial pulmonary valve implantation had been perfomed initially. Type I PA/IVS refers to mild dysplasia of the right ventricle; the tricuspid valve and right ventricular cavity reach 2/3 above normal, and the right ventricular outflow tract is well developed. Type II PA/IVS refers to moderate dysplasia of the right ventricle; the tricuspid valve and right ventricular cavity reach normal 1/3–2/3, and the right ventricular outflow tract is well developed. All patients had patent ductus arteriosus (PDA), atrial septal defect (ASD)/patent foramen ovale (PFO) and extensive tricuspid regurgitation (TR) (Table 1). The study was approved by the Ethics Committee of the Second Hospital of Hebei Medical University and conformed to the principles of the Helsinki declaration. The requirement for informed consent was waived because of the retrospective nature of the study.

**Table 1:** Basic condition of children before surgery

Group	Age	Male/female	Height	Weight	Tad	Tz	McGoon	SpO2	PDA	ASD/Pfo	TR(++)
A	24.04 ± 14.36	14(56%)11(44%)	52.42 ± 2.33	3.35 ± 0.25	9.32 ± 0.71	− 1.63 ± 0.30	1.03 ± 0.09	0.71 ± 0.03	2. 28 ± 0.39	2.92 ± 0.29	25(++)
B	25.48 ± 12.71	12(48%)13(52%)	51.41 ± 1.97	3.29 ± 0.18	9.57 ± 0.75	− 1.43 ± 0.39	1.05 ± 0.08	0.69 ± 0.04	2.61 ± 0.35	3.12 ± 0.36	25(++)
C	37.28 ± 7.51	14(56%)11(44%)	52.20 ± 0.83	3.49 ± 0.19	7.01 ± 0.57	− 3.28 ± 0.36	0.95 ± 0.04	0.65 ± 0.03	2.53 ± 0.36	2.78 ± 0.24	25(++)
D	34.40 ± 8.59	9(36%)16(64%)	52.08 ± 1.17	3.43 ± 0.16	6.84 ± 0.44	− 3.31 ± 0.35	0.98 ± 0.05	0.68 ± 0.05	2.68 ± 0.28	2.88 ± 0.34	25(++)
Pab	0.62	1.0	0.55	0.06	0.41	0.16	0.73	0.17	0.67	0.28	–
Pcd	0.33	0.16	0.10	0.20	0.26	0.99	0.42	0.09	0.25	0.31	–

Tad: Tricuspid annulus diameter Tz: Tricuspid Z value TR: Tricuspid regurgitation Pab: *P* value between group A and group B Pcd: *P* value between group C and group D (++): massive tricuspid valve regurgitation A: group A (type I PA/IVS) with BT shunt + PDA ligation + balloon dilatation of pulmonary valve. B: groupB (type I PA/IVS) with BT shunt + PDA ligation + pulmonary valve incision. C: groupC (type II PA/IVS) with BT shunt + PDA ligation + RVOT incision + transannular patch. D: group D (type II PA/IVS) with BT shunt + PDA ligation + RVOT incision, + transannular patch + artificial pulmonary valve implantation

### Preoperative preparation

Three echocardiographers with at least 10 years of working experience jointly performed echocardiography in children with PA/IVS. Finally, a comprehensive conclusion was given. Moreover, diagnostic cardiac catheterization and angiocardiography had been performed in 36 patients. The inner diameter of the tricuspid valve attached to the right ventricular wall was measured mainly through the four-chamber view of the heart. The Z value of tricuspid valve was obtained according to the one corresponding to different height, weight and body surface area, so as to evaluate the right ventricular development in children. The tricuspid Z value (− 2–0) indicated mild right ventricular dysplasia, and the one (− 4–2) indicated moderate right ventricular dysplasia. In addition, the ratio of tricuspid valve diameter to mitral valve diameter measured by echocardiography can provide some evidence of right ventricular development as a reference index. The diameter ratio of tricuspid valve to mitral valve in neonates was less than 0.7, suggesting right ventricular dysplasia. All patients were diagnosed as type I PA/IVS or type II PA/IVS without RVDCC. There were no significant differences between groups A and B, and between groups C and D with respect to gender, age, weight, tricuspid Z value, degree of TR, McGoon ratio, oxygen saturation (SpO2), inner diameter of PDA, size of ASD or PFO (all *P* > 0.05, Table 1).

### Surgical methods

**Fig. 1:**
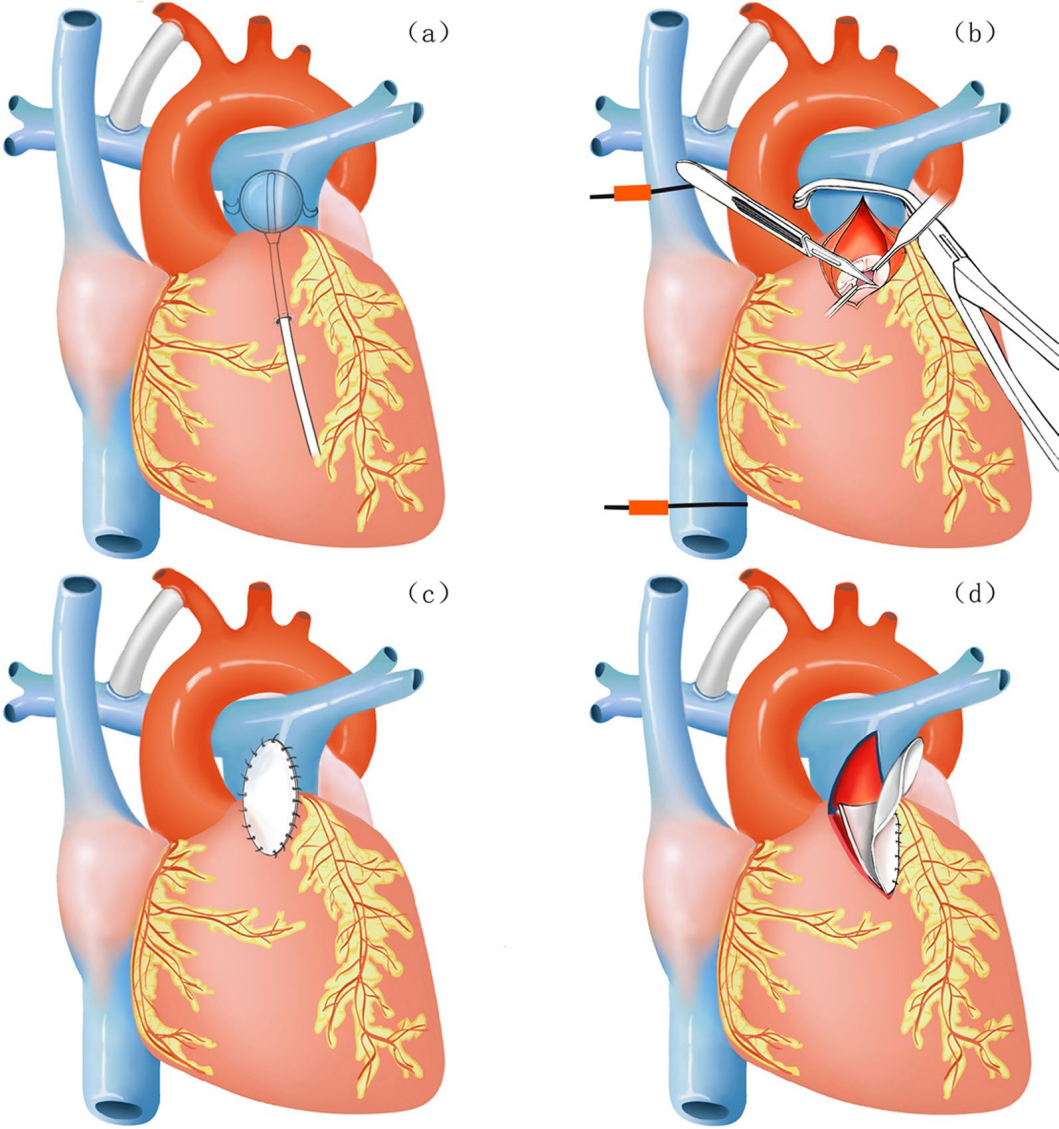
Graphics of four surgical procedures. **a** Group A (type I PA/IVS) with BT shunt + PDA ligation + balloon dilatation of pulmonary valve. **b** Group B (type I PA/IVS) with BT shunt + PDA ligation + pulmonary valve incision. **c** Group C (type II PA/IVS) with BT shunt + PDA ligation + RVOT incision + transannular patch. **d** Group D (type II PA/IVS) with BT shunt + PDA ligation + RVOT incision, + transannular patch + artificial pulmonary valve implantation

The initial surgery had been performed in group A with BT shunt combined with PDA ligation and balloon dilatation of pulmonary valve, and in group B with BT shunt combined with PDA ligation and pulmonary valve incision. Group C had been treated with BT shunt combined with PDA ligation, right ventricular outflow tract (RVOT) incision and transannular patch, and group D had undergone BT shunt combined with PDA ligation, RVOT incision, transannular patch and artificial pulmonary valve implantation (Fig. 1). BT shunt was performed by setting up a stretch vascular graft (W.L.Gore & Associates, Inc., 1505 North Fourth Street Flagstaff Arizona USA) between the brachiocephalic artery and the right pulmonary artery. According to the experience of our department, the type of the stretch vascular graft was selected based on the weight of the children. The 3 mm stretch vascular graft is recommended when the weight is less than 2.5 kg. The stretch vascular graft with a diameter of 3.5 mm is preferable for the newborn and within 3 kg, and the 4 mm stretch vascular graft is recommended for 3–7 kg. In order to avoid hypoxia intolerance, PDA was ligated after BT shunt, which could stabilize blood SpO2, and avoid blood flow conflicts from the right ventricle to PDA. Balloon dilatation of pulmonary valve, which had achieved good clinical results [4], was as follows: Under direct vision, the pulmonary catheter was inserted into the pulmonary valve, and the pulmonary artery anulus was expanded under the guidance of transesophageal echocardiography (TEE). According to our experience, the expansion is safer when the ratio of balloon diameter to pulmonary valve annulus diameter is 1:1.3. The changes of pulmonary artery pressure were observed immediately by TEE after balloon dilatation. If the pressure difference was still greater than 30 mm Hg, the balloon tube 1–2 times larger than the initial expansion balloon was selected for repeated expansion. Pulmonary valve incision was performed without extracorporeal circulation, that is, temporary occlusion of superior vena cava, inferior vena cava and distal main pulmonary artery, open atresia of pulmonary valve through main pulmonary artery incision under direct vision. Since the right ventricular and tricuspid valve development of type II PA/IVS is worse than that of type I PA/IVS, it is necessary to cut the right ventricular outflow tract and pulmonary valve annulus and have transvalvular patch to further reduce the pressure of the right ventricular cavity and TR, so that the right ventricular cavity and tricuspid annulus will have growth potential. Artificial single pulmonary valve, made of 0.1 mm thick pericardial membrane (W.L.Gore & Associates, Inc., 1505 North Fourth Street Flagstaff Arizona USA) by cutting into the shape of the pulmonary valve and being sutured to the transvalvular patch near the pulmonary valve annulus, was used according to the diameter of pulmonary valve anulus obtained by body surface area of children, so as to minimize pulmonary valve regurgitation.

Four groups of patients underwent the initial surgery according to the corresponding surgical methods. The mechanical ventilation time, length of ICU stay and perioperative mortality rate were recorded and statistically analyzed. The tricuspid Z value, McGoon ratio, and TR in each group were recorded before surgery, and at 1 month, 3 months, 6 months, 12 months, 24 months, 36 months, 48 months, 60 months and 72 months after surgery, and statistical analysis was performed. Pulmonary regurgitation (PR) and pulmonary artery transvalvular pressure were recorded and statistically analyzed before discharge, and at 1 month, 3 months, 6 months 12 months, 24 months, 36 months, 48 months, 60 months and 72 months after surgery. During the follow-up period, the data of patients who had undergone surgical intervention or radical surgery were excluded at the corresponding time points. The survival rate from the day of surgery to 72 months after initial surgery were recorded and statistically analyzed.

### Statistical analysis

IBM SPSS Statistics22 statistical software was used for statistical analysis. One-sample Kolmogorov–Smirnov Test was used to determine normality of data. Measurement data are expressed as mean ± standard deviation (${\overline{\text{x}}}$ ± s). Differences in categorical variables between groups were tested using independent sample t test, Pearson chi-square test, and Continuity Correction. By R4.1.0 software, survival and survminer packages were used to build cox regression models and draw survival analysis curves. *P* < 0.05 indicated that the difference was statistically significant, and *P* < 0.01 indicated that the difference was highly significant.

## Results

**Fig. 2:**
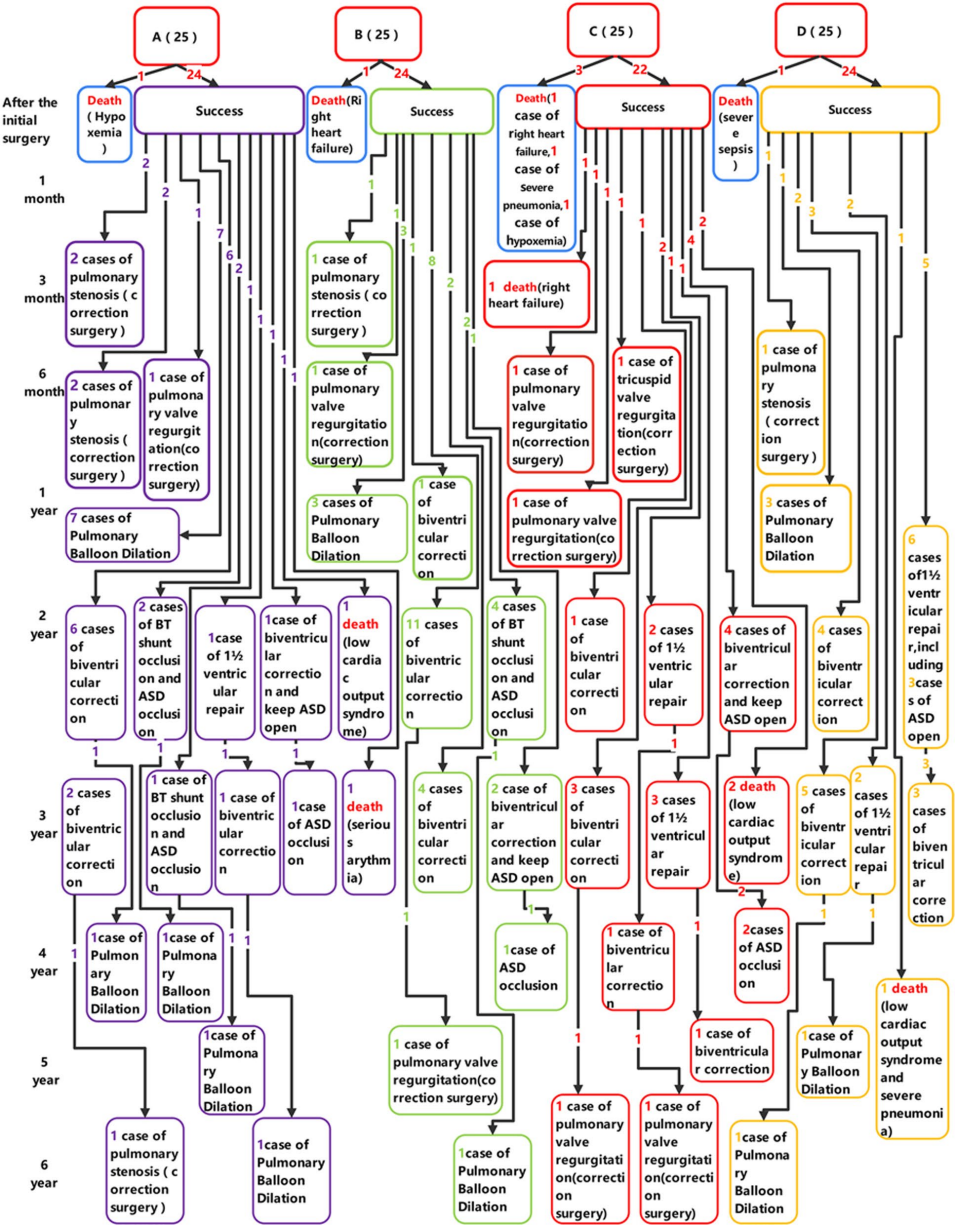
Flow chart displaying short-term outcomes of patients of type I and II PA/IVS with Different Initial Surgical Methods. **A** Group A (type I PA/IVS) with BT shunt + PDA ligation + balloon dilatation of pulmonary valve. **B** Group B (type I PA/IVS) with BT shunt + PDA ligation + pulmonary valve incision. **C** Group C (type II PA/IVS) with BT shunt + PDA ligation + RVOT incision + transannular patch. **D** Group D (type II PA/IVS) with BT shunt + PDA ligation + RVOT incision, + transannular patch + artificial pulmonary valve implantation

One case died of hypoxemia after the initial surgery in group A (Fig. 2); at 3 months after surgery, 2 cases underwent reoperation due to pulmonary stenosis (Fig. 2); 1 case was re-operated due to pulmonary valve regurgitation and 2 cases underwent reoperation due to pulmonary stenosis at 6 months after the initial surgery (Fig. 2); 7 cases were treated with pulmonary balloon dilation at 12 months after surgery, (Fig. 2); at 24 months after the initial surgery, 6 cases successfully underwent biventricular correction, 2 cases were treated with BT shunt occlusion and ASD occlusion, 1 case was re-operated with 1½ ventricular repair, 1 case underwent biventricular correction and keeping ASD open, and 1 case died of low cardiac output syndrome (Fig. 2); 1 case died of serious arythmia, 3 cases successfully underwent biventricular correction, 1 case was treated with BT shunt occlusion and ASD occlusion, and 1 case was re-operated with ASD occlusion at 36 months after surgery (Fig. 2); at 48 months after surgery, 2 cases underwent pulmonary balloon dilation (Fig. 2); 1 case was re-operated with pulmonary balloon dilation at 60 months after surgery (Fig. 2); at 72 months after surgery, 1 case underwent reoperation due to pulmonary stenosis, and 1 case was retreated with pulmonary balloon dilation (Fig. 2). In group B, 1 case died of right heart failure after the initial surgery, and there was 1 case of pulmonary stenosis who received reoperation at 3 months after surgery (Fig. 2); 1 case was re-operated due to pulmonary valve regurgitation at 6 months after the initial surgery (Fig. 2); 1 case successfully underwent biventricular correction, and 1 case was treated with pulmonary balloon dilation at 12 months after the initial surgery (Fig. 2); at 24 months after surgery, 11 cases successfully underwent double ventricular correction, and 4 cases were treated with BT shunt occlusion and ASD occlusion (Fig. 2); 4 cases underwent biventricular correction, and 2 cases were treated with biventricular correction and keeping ASD open at 36 months after surgery (Fig. 2); at 48 months after surgery, 1 case underwent ASD occlusion (Fig. 2); 1 case was re-operated due to pulmonary valve regurgitation at 60 months after surgery (Fig. 2); at 72 months after surgery, there was 1 case of pulmonary stenosis who underwent pulmonary balloon dilation (Fig. 2). In group C, 3 cases died after surgery, including 1 case who died of right heart failure, 1 case who died of severe pneumonia and 1 case who died of hypoxemia (Fig. 2); 1 case with pulmonary valve regurgitation and 1 case with TR were re-operated at 6 months after the initial surgery, and there was 1 case who had reoperation due to pulmonary valve regurgitation at 1 year after surgery (Fig. 2); at 24 months after surgery, 1 case successfully underwent biventricular correction, 2 cases were treated with 1½ ventricular repair, and 4 cases were re-operated by biventricular correction and keeping ASD open (Fig. 2); 2 cases died of low cardiac output syndrome at 36 months after surgery, and 3 cases were successfully treated with biventricular correction, 2 cases underwent 1½ ventricular repair; at 48 months after surgery, 1 case underwent biventricular correction, and 2 cases were treated with ASD occlusion (Fig. 2); 1 case was successfully treated with biventricular correction at 60 months after surgery (Fig. 2); 2 cases were re-operated due to pulmonary valve regurgitation at 72 months after surgery. In group D, 1 case died of severe sepsis after the initial surgery, 1 case with pulmonary stenosis had correction surgery at 6 months after surgery, and 3 cases was re-operated by pulmonary balloon dilation at 1 year after the initial surgery (Fig. 2); at 24 months after surgery, 4 cases successfully underwent biventricular correction, 6 cases were treated with 1½ ventricular repair, including 4 cases with ASD open (Fig. 2); 8 cases were successfully treated with biventricular correction, and 2 cases underwent 1½ ventricular repair at 36 months after surgery (Fig. 2); at 60 months after surgery, 1 case died of low cardiac output syndrome and severe pneumonia, 1 case was re-operated by pulmonary balloon dilation (Fig. 2); there was 1 case of pulmonary stenosis who underwent pulmonary balloon dilation at 72 months after surgery (Fig. 2).

**Fig. 3:**
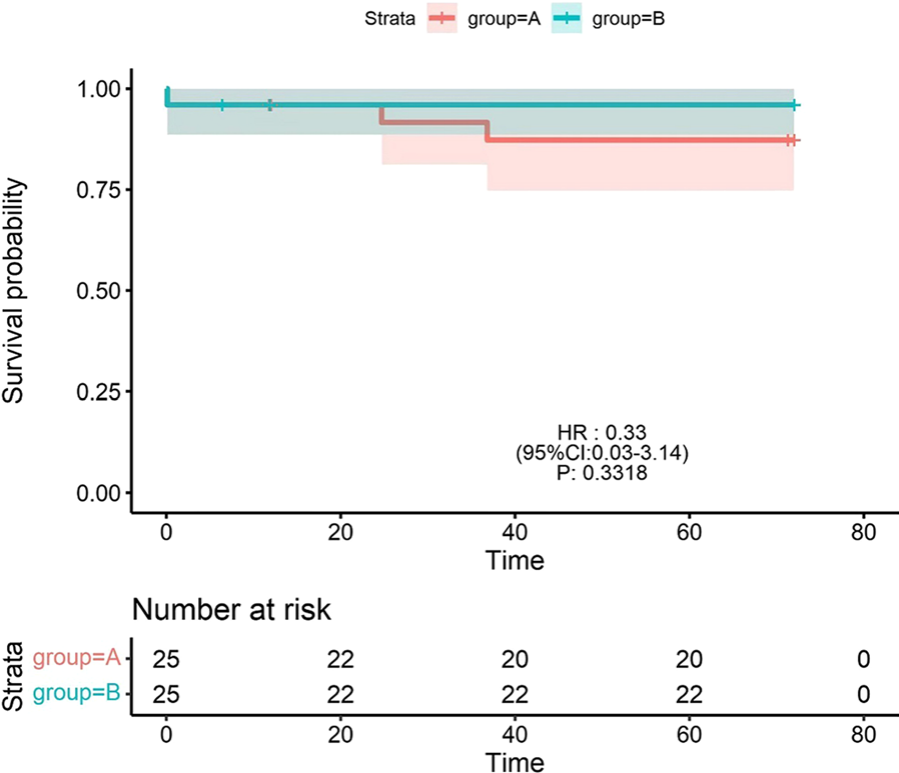
Survival analysis curves between group A and group B plot by R4.1.0 software. Group A (type I PA/IVS) with BT shunt + PDA ligation + balloon dilatation of pulmonary valve. Group B (type I PA/IVS) with BT shunt + PDA ligation + pulmonary valve incision

**Fig. 4:**
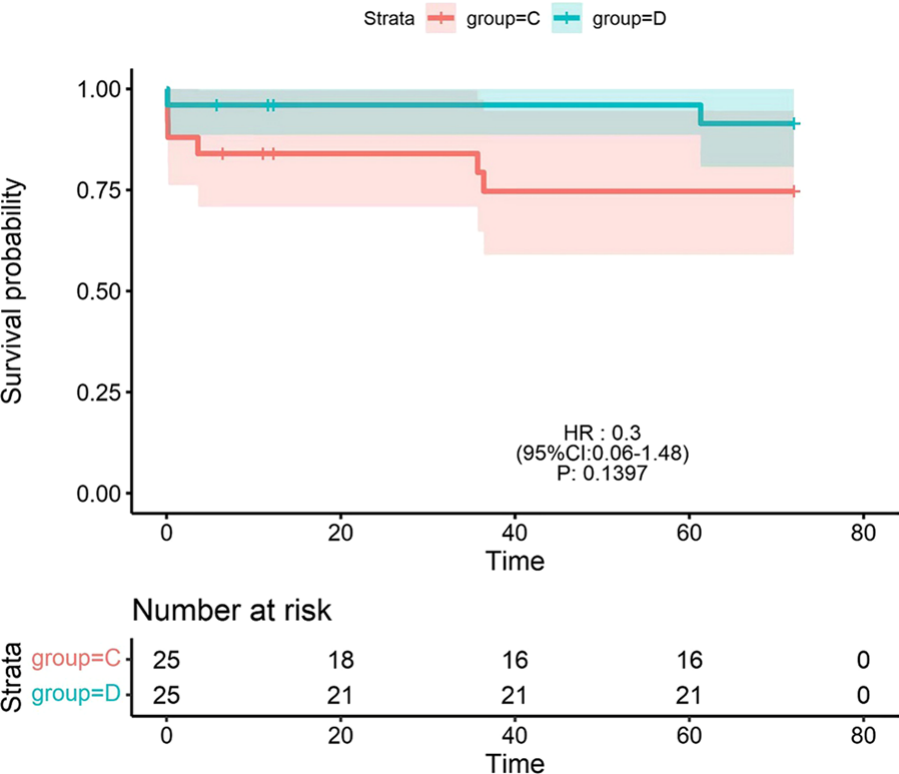
Survival analysis curves between group C and group D plot by R4.1.0 software. Group C (type II PA/IVS) with BT shunt + PDA ligation + RVOT incision + transannular patch; group D (type II PA/IVS) with BT shunt + PDA ligation + RVOT incision, + transannular patch + artificial pulmonary valve implantation

There were no statistically significant differences in the mechanical ventilation time, length of ICU stay, and perioperative mortality rate after surgery between group A and group B (all *P* > 0.05; Table 2). The PR was significantly greater in group A than in group B at 1 month after surgery (*P* < 0.05; Table 3). At 3 months after surgery, the tricuspid Z value and McGoon ratio were lower in group A than in group B (*P* < 0.05; Table 3), whereas the PR, pulmonary artery transvalvular pressure, and TR were significantly greater in group A than in group B (*P* < 0.05; Table 3). At 6 months after surgery, the SpO2 was lower in group A than in group B (*P* < 0.05; Table 3), while the pulmonary artery transvalvular pressure was significantly greater in group A than in group B (*P* < 0.01; Table 3). The pulmonary artery transvalvular pressure was greater in group A than in group B at 12 months after surgery (*P* < 0.05; Table 3). At 24 months after surgery, the tricuspid Z value and McGoon ratio were lower in group A than in group B (*P* < 0.05; Table 3), whereas the pulmonary artery transvalvular pressure was significantly greater in group A than in group B (*P* < 0.05; Table 3). At 36 months after surgery, the pulmonary artery transvalvular pressure was significantly greater in group A than in group B (*P* < 0.05; Table 3). There was no statistically significant difference in the survival rate between group A and group B (*P* > 0.05; Fig. 3). Mechanical ventilation time and length of ICU stay were greater in group C than in group D (*P* < 0.05; Table 2). At 1 month after surgery, the PR was significantly higher in group C than in group D (*P* < 0.01; Table 3); at 3 months after surgery, the PR in group C was higher than those in group D (*P* < 0.05; Table 3). The McGoon ratio, SpO2 were lower in group C than in group D (*P* < 0.05; Table 3), whereas the TR in group C was higher than those in group D (*P* < 0.05; Table 3). At 6 months after surgery, the PR was higher in group C than in group D (*P* < 0.05; Table 3), while the tricuspid Z value and pulmonary artery transvalvular pressure were lower than those in group D (*P* < 0.05; Table 3). At 12 months after surgery, the pulmonary artery transvalvular pressure was significantly higher in group D than in group C (*P* < 0.01; Table 3), and at 36 months after surgery, the pulmonary artery transvalvular pressure was significantly higher in group D than in group C (*P* < 0.05; Table 3). There was no significant difference in survival rate between group C and group D (*P* > 0.05; Fig. 4).

**Table 2:** Mechanical ventilation time, length of ICU stay, mortality rate

Group	MVT	ICU	Mortality rate
A	37.76 ± 3.67	151.16 ± 10.19	1(4%)
B	39.52 ± 3.53	158.88 ± 11.08	1(4%)
C	80.96 ± 8.42	222.00 ± 11.72	3(12%)
D	65.16 ± 4.85	162.48 ± 7.91	1(4%)
Pab	0.83	0.39	1.00
Pcd	0.045	0.048	0.60

MVT: mechanical ventilation time, ICU: length of ICU stay, Pab: *P* value between group A and group B, Pcd: *P* value between group C and group D. A: group A (type I PA/IVS) with BT shunt + PDA ligation + balloon dilatation of pulmonary valve. B: group B (type I PA/IVS) with BT shunt + PDA ligation + pulmonary valve incision. C: group C (type II PA/IVS) with BT shunt + PDA ligation + RVOT incision + transannular patch. D: group D (type II PA/IVS) with BT shunt + PDA ligation + RVOT incision, + transannular patch + artificial pulmonary valve implantation

**Table 3:** Postoperative related indicators

Time	Group	Spo2	PTP	Mc	PR	TR	TZ
Discharge	A	0.77 ± 0.03	33.88 ± 3.04		22(++)2(+)		
	B	0.80 ± 0.02	32.13 ± 2.80		19(++)5(+)		
	C	0.73 ± 0.02	19.50 ± 2.69		21(++)1(+)		
	D	0.75 ± 0.03	22.79 ± 2.50		10(++)10(+)		
	Pab	0.43	0.69		0.41		
	Pcd	0.10	0.56		0.001		
1 month	A	0.78 ± 0.05	49.17 ± 2.65	1.04 ± 0.07	22(++)2(+)	19(++)5(+)	− (1.62 ± 0.17)
	B	0.81 ± 0.04	47.46 ± 3.40	1.05 ± 0.09	16(++)8(+)	18(++)6(+)	− (1.42 ± 0.19)
	C	0.75 ± 0.04	22.05 ± 2.38	0.94 ± 0.06	21(++)1(+)	20(++)2(+)	− (3.39 ± 0.17)
	D	0.77 ± 0.03	23.67 ± 2.06	0.98 ± 0.07	10(++)14(+)	17(++)7(+)	− (3.36 ± 0.16)
	Pab	0.22	0.30	0.38	0.03	0.73	0.70
	Pcd	0.71	0.42	0.62	0.00	0.18	0.89
3 month	A	0.80 ± 0.04	64.86 ± 4.13	1.06 ± 0.06	18(++)6(+)	19(++)5(+)	− (1.43 ± 0.06)
	B	0.84 ± 0.03	53.04 ± 5.64	1.21 ± 0.04	11(++)13(+)	12(++)12(+)	− (1.37 ± 0.04)
	C	0.81 ± 0.02	30.38 ± 2.80	0.97 ± 0.05	17(++)4(+)	18(++)3(+)	− (3.28 ± 0.08)
	D	0.83 ± 0.01	33.42 ± 3.35	1.15 ± 0.07	11(++)13(+)	14(++)10(+)	− (3.16 ± 0.07)
	Pab	0.30	0.045	0.04	0.04	0.04	0.03
	Pcd	0.02	0.61	0.04	0.02	0.04	0.58
6 month	A	0.87 ± 0.02	69.47 ± 1.93	1.17 ± 0.05	14(++)10(+)	10(++)14(+)	− (1.31 ± 0.04)
	B	0.88 ± 0.04	55.95 ± 4.04	1.23 ± 0.04	10(++)14(+)	11(++)13(+)	− (1.28 ± 0.07)
	C	0.85 ± 0.02	37.84 ± 3.10	1.09 ± 0.07	19(++)2(+)	12(++)9(+)	− (3.15 ± 0.07)
	D	0.87 ± 0.03	43.43 ± 4.28	1.17 ± 0.05	15(++)9(+)	9(++)15(+)	− (2.78 ± 0.04)
	Pab	0.02	0.005	0.22	0.25	0.77	0.18
	Pcd	0.29	0.044	0.06	0.03	0.19	0.03
12 month	A	0.91 ± 0.03	80.16 ± 3.76	1.21 ± 0.04	9(++)15(+)	8(++)16(+)	− (1.28 ± 0.04)
	B	0.93 ± 0.03	73.24 ± 2.34	1.24 ± 0.03	8(++)16(+)	9(++)15(+)	− (1.20 ± 0.03)
	C	0.91 ± 0.03	53.86 ± 3.63	1.20 ± 0.03	18(++)3(+)	8(++)13(+)	− (2.56 ± 0.08)
	D	0.92 ± 0.04	66.95 ± 7.66	1.21 ± 0.05	19(++)5(+)	6(++)18(+)	− (2.43 ± 0.07)
	Pab	0.95	0.035	0.63	0.55	0.76	0.82
	Pcd	0.99	0.009	0.11	0.86	0.34	0.07
24 month	A	0.94 ± 0.01	62.95 ± 5.64	1.39 ± 0.18	5(++)17(+)	5(++)17(+)	− (0.51 ± 0.11)
	B	0.95 ± 0.01	48.47 ± 7.44	1.48 ± 0.12	6(++)16(+)	4(++)18(+)	− (0.41 ± 0.06)
	C	0.91 ± 0.01	50.86 ± 1.68	1.31 ± 0.09	11(++)7(+)	4(++)14(+)	− (1.09 ± 0.13)
	D	0.93 ± 0.01	56.86 ± 3.91	1.33 ± 0.07	9(++)12(+)	5(++)16(+)	− (0.99 ± 0.11)
	Pab	0.73	0.04	0.04	0.73	1.0	0.02
	Pcd	0.79	0.21	0.27	0.34	1.0	0.24
36 month	A	0.95 ± 0.01	53.69 ± 4.89	1.70 ± 0.13	4(++)17(+)	5(++)16(+)	0.05 ± 0.05
	B	0.96 ± 0.01	45.77 ± 3.26	1.78 ± 0.09	4(++)18(+)	3(++)19(+)	0.06 ± 0.05
	C	0.93 ± 0.01	43.32 ± 4.09	1.45 ± 0.09	6(++)12(+)	4(++)14(+)	− (0.45 ± 0.08)
	D	0.94 ± 0.01	45.48 ± 2.47	1.49 ± 0.13	5(++)16(+)	4(++)17(+)	− (0.37 ± 0.07)
	Pab	0.68	0.02	0.14	1.0	0.64	0.80
	Pcd	0.61	0.03	0.10	0.72	1.0	0.60
48 month	A	0.96 ± 0.01	32.09 ± 2.42	1.79 ± 0.12	4(++)16(+)	4(++)16(+)	0.05 ± 0.04
	B	0.97 ± 0.01	24.76 ± 2.34	1.91 ± 0.14	3(++)19(+)	4(++)18(+)	0.07 ± 0.06
	C	0.94 ± 0.01	31.07 ± 1.88	1.66 ± 0.08	4 (++)12(+)	3(++)12(+)	0.04 ± 0.07
	D	0.95 ± 0.02	34.58 ± 2.21	1.68 ± 0.11	5(++)16(+)	4(++)17(+)	0.05 ± 0.06
	Pab	0.47	0.82	0.93	0.89	1.0	0.09
	Pcd	0.19	0.57	0.25	1.0	1.0	0.89
60 month	A	0.95 ± 0.01	24.22 ± 2.20	1.94 ± 0.10	3(++)17(+)	4(++)16(+)	0.08 ± 0.04
	B	0.96 ± 0.01	22.51 ± 1.95	1.96 ± 0.13	3(++)19(+)	3(++)19(+)	0.10 ± 0.07
	C	0.95 ± 0.01	24.62 ± 1.90	1.84 ± 0.08	4(++)13(+)	3(++)13(+)	0.07 ± 0.05
	D	0.96 ± 0.01	28.37 ± 2.13	1.85 ± 0.10	4(++)17(+)	3(++)18(+)	0.09 ± 0.07
	Pab	0.46	0.39	0.24	1.0	0.89	0.14
	Pcd	0.29	0.62	0.41	1.0	1.0	0.45
72 month	A	0.95 ± 0.01	21.96 ± 2.24	1.93 ± 0.12	3(++)16(+)	2(++)19(+)	0.09 ± 0.05
	B	0.96 ± 0.01	20.65 ± 1.79	1.91 ± 0.14	3(++)19(+)	2(++)20(+)	0.11 ± 0.06
	C	0.95 ± 0.01	20.67 ± 1.59	1.90 ± 0.12	3(++)13(+)	3(++)13(+)	0.08 ± 0.05
	D	0.96 ± 0.01	25.12 ± 1.90	1.89 ± 0.15	3(++)17(+)	2(++)18(+)	0.09 ± 0.06
	Pab	0.76	0.21	0.63	1.0	1.0	0.50
	Pcd	0.54	0.20	0.24	0.68	0.64	0.27

Spo2: Oxygen saturation, PTP: Pulmonary artery transvalvular pressure, PR: Pulmonary valve regurgitation, TR: Tricuspid valve regurgitation, TZ: Tricuspid valve Z value, Mc: McGoon ratio Discharge: the day of discharge, 1 month:1 month after surgery, 3 month:3 months after surgery, 6 month:6 months after surgery, 1 year:1 year after surgery, 2 year:2 year after surgery, 3 year:3 year after surgery, 4 year:4 year after surgery, 5 year:5 year after surgery, 6 year:2 year after surgery, Pab: *P* value between group A and group B, Pcd: *P* value between group C and group D, +  + : massive tricuspid/pulmonary valve regurgitation, + : minor tricuspid/pulmonary valve regurgitation. A: group A (type I PA/IVS) with BT shunt + PDA ligation + balloon dilatation of pulmonary valve. B: group B (type I PA/IVS) with BT shunt + PDA ligation + pulmonary valve incision. C: group C (type II PA/IVS) with BT shunt + PDA ligation + RVOT incision + transannular patch. D: group D (type II PA/IVS) with BT shunt + PDA ligation + RVOT incision, + transannular patch + artificial pulmonary valve implantation

## Discussion

The purpose of this study was to investigate the effects of two different kinds of initial surgical strategies on type I and II PA/IVS respectively, which has a low incidence but a high mortality rate and great anatomical variation. Surgical programs are also diverse in each center, therefore, there is still no unified standard. Individualized surgical treatment is favourable, and the key step is to improve the early survival rate. According to our experience, few patients have one-stage radical operation; instead, most need staging surgery. Moreover, it is important to pay attention to the anatomy of the coronary arteries. The ratio of right ventricular-coronary artery fistula is higher if echocardiography shows severe right ventricular dysplasia and narrow tricuspid valve opening, usually in which there is coronary artery intimal fibrous hyperplasia, resulting in coronary artery stenosis or even complete occlusion. RVDCC could be caused if obstruction occurs at the proximal end of the coronary artery. Such children have a high risk of myocardial ischemia, that is, hypoxic blood is perfused from the right ventricle to a considerable range of myocardium, even worse when the diastolic aortic pressure falls after BT shunt is established. It is hard for such children to tolerate the drop in right ventricular pressure if right ventricular outflow tract patch and pulmonary valve incision are performed to implement right ventricular decompression, and acute myocardial infarction will occur. In this study, the children’s right ventricle and tricuspid valve development are mild to moderate dysplasia, and even some children had undergone cardiac catheterization before surgery, which can rule out coronary artery malformations. For patients with mild and moderate right ventricular dysplasia and without RVDCC, right ventricular decompression should be performed as early as possible through initial surgery, thereby promoting the development of the right ventricle, providing a precise pulmonary blood supply to improve the systemic arterial blood oxygen saturation, and improving the opportunity of biventricular repair in children [5, 6], and eventually completing biventricular repair as early as possible [7].

Although much more surgical methods of right ventricular decompression are available for PA/IVS [4, 8–12], surgical failure [13] and complications [14–17] are also universal. Hypoxemia is one of the most common complications for the following reasons: (1) SpO2 in the early stage is not well maintained because of muscle edema of the right ventricle; (2) Hypoplastic right ventricle is not capable of maintaining the sufficient forward blood flow after the opening of pulmonary valve [18], and the pulmonary vascular bed pressure that has not been fully decreased in the neonatal period also limits the blood flow.

BT shunt can be established to correct hypoxemia. Nonetheless, it is still controversial whether decompression surgery is routinely combined with BT shunt, and some studies have reported that BT shunt may lead to volume overload and left heart failure [19]. However, our study believes that PDA is more likely to close when SpO2 becomes higher after right ventricular decompression, which has a great effect on the prognosis of PA/IVS. Therefore, PDA is routinely ligated and BT shunt surgery is supplemented to ensure the stability and persistence of blood between systemic and pulmonary circulation.

In recent years, balloon dilatation of pulmonary valve has become a new approach for right ventricular decompression in the early stage of PA/IVS [4], which avoids the damage caused by cardiopulmonary bypass, with fast postoperative recovery, and could shorten the length of ICU stay. Pulmonary valve incision, which needs occlusion of superior and inferior vena cava and pulmonary artery temporarily, is a simple procedure, with quick postoperative recovery. This procedure has two advantages as follows: (1) Cardiopulmonary bypass is not required. (2) More accurate incision of atresia pulmonary valve can be done under direct vision. Compared with group A, group B also had achieved good surgical results, and there were no differences in mechanical ventilation time, length of ICU stay, and perioperative mortality rate (*P* > 0.05; Table 2). In this study, we establish the right ventricle-pulmonary artery forward blood flow through balloon dilatation of pulmonary valve or pulmonary valve incision in children with Type I PA/IVS.

However, the balloon dilatation of pulmonary valve has several shortcomings, in which the puncture needle may be deviated from the center of the pulmonary valve, and the pulmonary valve is unevenly dilated, which finally reduces the function of the pulmonary valve and causes severe PR. Moreover, due to tissue growth, adhesion, scar and other reasons after balloon dilatation, pulmonary valve will be reduced to varying degrees, hindering the growth of the right ventricle.

The corresponding data showed that the PR was greater in group A than in group B at 1 and 3 months after surgery (*P* < 0.05; Table 3), and the pulmonary transvalvular pressure was higher in group A than in group B at 3 months (*P* < 0.05; Table 3), 6 months (*P* < 0.01; Table 3), 12 months (*P* < 0.05; Table 3), 24 months (*P* < 0.05; Table 3) and 36 months after surgery (*P* < 0.05; Table 3). In conclusion, pulmonary valve incision is better and more economical than balloon dilatation of pulmonary valve, since it does not have the forementioned disadvantages, nor use intervention consumables, such as pulmonary artery balloon.

Compared with group C, group D added an artificial pulmonary valve made of Gore-tex membrane, which could reduce pulmonary valve regurgitation, protect right ventricular function, shorten mechanical ventilation time and length of ICU stay, promote right ventricular development, and improve the survival rate of initial surgery to a certain extent. The corresponding data showed that the PR was higher in group C than in group D at discharge (*P* < 0.05; Table 3), and at 1 month (*P* < 0.01; Table 3), 3 months (*P* < 0.05; Table 3), and 6 months after surgery (*P* < 0.05; Table 3). The mechanical ventilation time and length of ICU stay were longer in group C than in group D, (*P* < 0.05; Table 2). As for the causes of death in groups C and D, right heart failure was the highest in group C, which also indicated the advantages of artificial pulmonary valve in protecting the right ventricle.

Since each surgical method has its advantages and disadvantages, and different surgeons have different understandings and preferences for the same surgical method. Meanwhile, the selection of surgical methods also should be combined with the overall state of children for comprehensive evaluation. For example, if children with type I PA/IVS would performed with pulmonary valve incision, superior vena cava, inferior vena cava and distal main pulmonary artery need to be temporarily occluded. If the vital signs are not stable and children can not tolerate this operation, only pulmonary balloon dilatation can be selected. For children with type II PA/IVS, especially with membranous atresia, artificial pulmonary valve was probably unnecessary if the remaining pulmonary valve membrane was in good condition,. If there were calcification and stiffness in the pulmonary valve, artificial pulmonary valve could be made to reduce pulmonary blood reflux. However, due to the presence of calcified contracture and lack of growth in the artificial pulmonary valve, complications such as pulmonary artery stenosis and obstruction can be caused in the postoperative period. The corresponding data showed that the pulmonary artery transvalvular pressure was greater in group D than in group C at 6 month (*P* < 0.05; Table 3), 12 months (*P* < 0.01; Table 3), and 36 months after surgery (*P* < 0.05; Table 3). Moreover, some children have good tolerance to pulmonary regurgitation. Therefore, whether to add artificial pulmonary valve also need to be considered individually comprehensively.

In addition, this study has several limitations. First, we failed to detect the real differences between groups due to insufficient sample size and low incidence. Second, the follow-up period after initial surgery was insufficient to reflect the trend of the observation indexes, which may have introduced a certain bias on the statistical analysis results. Furthermore, several patients with radical conditions did not receive timely surgery, and some patients who needed early reintervention fail to undergo timely surgery for various reasons, including large pulmonary transvalvular pressure or severe PR. It was related to the economic conditions and ideas of the guardians of the patients. Some parents could not afford medical treatment, and some parents believed that their children were in good living conditions and did not need reoperation. Moreover, it is also associated with the conception of several surgeons that the older the child is, the higher the success rate of reoperation is.

## Conclusion

Early active surgical treatment of PA/IVS is the key to improving the survival rate, and staged decompression is relatively stable, even if this may increase the rate of postoperative reintervention. The reasonable choice of the initial surgical strategy is an important factor affecting the prognosis. Taken together, BT shunt combined with PDA ligation and pulmonary valve incision is superior to BT shunt combined with PDA ligation and balloon dilatation of pulmonary valve, and BT shunt combined with PDA ligation, RVOT incision, transannular patch and artificial pulmonary valve implantation is superior to BT shunt combined with PDA ligation, RVOT incision and transannular patch.

## Data Availability

The datasets used and analyzed during the current study are available from the corresponding author on reasonable request.
